# Th2 predominance and decreased NK cells in patients with hereditary angioedema

**DOI:** 10.3389/fimmu.2025.1536128

**Published:** 2025-05-14

**Authors:** Linda Sundler Björkman, Evelina Elmér, Arne Egesten, Lillemor Skattum

**Affiliations:** ^1^ Respiratory Medicine, Allergology & Palliative Medicine, Department of Clinical Sciences Lund, Lund University and Skåne University Hospital, Lund, Sweden; ^2^ Division of Transfusion Medicine, Department of Laboratory Medicine, Lund University, Lund, Sweden; ^3^ Department of Clinical Immunology and Transfusion Medicine, Skåne University Hospital, Lund, Sweden; ^4^ Division of Microbiology, Immunology and Glycobiology, Department of Laboratory Medicine, Lund University, Lund, Sweden

**Keywords:** hereditary angioedema, adaptive immunity, NK cells, T helper 2 cells, complement activation

## Abstract

**Background:**

In this study we included patients with hereditary angioedema (HAE) caused by decreased levels of C1 inhibitor (HAE-C1INH). An increased risk of autoimmune disorders, particularly systemic lupus erythematosus (SLE), has been reported in HAE-C1INH. This suggests that complement consumption affects adaptive immunity.

**Objective:**

To investigate lymphocyte subpopulations in relation to disease activity and complement activation in HAE-C1INH patients and matched controls.

**Methods:**

Flow cytometry of peripheral blood lymphocyte populations, measurements of complement and complement fragments, and collection of clinical data.

**Results:**

NK cell counts were lower in HAE-C1INH patients, and their frequencies were related to disease activity. The T helper (Th) cell balance was skewed towards more Th2 cells and less Th1 cells in HAE-C1INH patients compared to controls. There were also lower frequencies of class-switched B cells and plasmablasts in patients. Levels of C4 and the complement activation fragment C3d were related to disease activity.

**Conclusions:**

Blood lymphocyte populations are altered in HAE-C1INH, a finding which may be of pathophysiological importance considering the increased risks of both autoimmunity and allergy associated with HAE-C1INH.

## 1 Introduction

Hereditary angioedema (HAE) is a rare genetic disease, caused by a deficiency (type 1) or defective function (type 2) of the C1 inhibitor (C1INH; HAE-C1INH), due to pathogenic variants in the *SERPING1* gene. Pathogenic variants in other genes can cause HAE with normal C1INH, for example in *F12*, *PLG* and *KNG*. In this study only patients with HAE-C1INH were included. The disease is characterized by attacks of subcutaneous and submucosal edema, most commonly affecting the skin, the gastrointestinal tract, and the larynx. C1INH is an important regulator of complement activation but also of the contact, coagulation, and fibrinolytic systems. The swellings are caused by increased production of bradykinin, resulting from activation of the contact system (1).

There are several studies pointing to a higher risk of autoimmune disease in patients with HAE-C1INH, particularly systemic lupus erythematosus (SLE) (2–5). Furthermore, autoreactive B cells have been found in HAE-C1INH (6). An increased risk of autoimmune disease in HAE-C1INH might be linked to chronic complement consumption in several ways. Complement consumption in patients with HAE-C1INH results in low levels of complement C4 and consequently, low, or absent function of the classical pathway. This in turn leads to increased risk of inefficient clearing of immune complexes and apoptotic material (defective efferocytosis), where potential autoantigens (e.g., nuclear proteins and DNA) are exposed, potentially leading to a break of tolerance and production of autoantibodies (7, 8). In addition, increased concentrations of circulating complement fragments resulting from complement activation may influence the regulation of both B and T cells (9).

The aim of this study was to investigate the adaptive immune system in HAE-C1INH patients in relation to disease activity and degree of complement activation, by characterizing lymphocyte populations, complement and complement activation biomarkers in patients and matched controls.

## 2 Materials and methods

### 2.1 Study design

The study was designed as a case-control study. Samples for all laboratory analyses were collected on the same occasion for each study person.

### 2.2 Study population

Sixteen adults with a diagnosis of HAE-C1INH were, after written informed consent was obtained, consecutively (2020-2022) included in the study at the Department of Respiratory Medicine and Allergy, Skåne University Hospital in Lund, Sweden. None of the patients were having angioedema attacks at the time of sampling. All the patients included had a confirmed diagnosis of HAE type 1, based on low levels of C4 and C1INH in at least two different samples. Also, all patients had a positive family history of angioedemas and/or a pathogenic variant in the *SERPING1* gene. Three of the HAE-C1INH patients had an autoimmune disease (ulcerative colitis, hypothyroidism and psoriasis, respectively), however none of them were treated with immunomodulatory drugs. Patient characteristics and demographic data are described in **Supplementary Table S1**. Healthy volunteers, without known autoimmune disease, cancer, or ongoing infection, and matched for age and sex, were used as controls after informed consent, for comparisons of lymphocyte populations and complement levels.

### 2.3 Laboratory analyses and clinical parameters

Platelets, white blood cell count (WBC) including neutrophils, eosinophils, basophils, lymphocytes, and monocytes were analyzed by clinical routine methods at the Department of Clinical Chemistry, Skåne University Hospital, Lund. Serum levels of C1INH, C3, C4, and EDTA plasma levels of C3d were analyzed by clinical routine methods at the Department of Clinical Immunology and Transfusion Medicine, Skåne University Hospital, Lund. C1INH values below the detection level of the method (<0.04 g/L) were set to 0.03 to allow for inclusion in statistical analyses. C3d values below the detection level of the method (<3.1 mg/L) were set to 3.0 mg/L. C4 values below the detection level of the method (<0.02 g/L) were set to 0.01 g/L.

The following complement fragments were analyzed using ELISA; C3a (cat. # A032; MicroVue C3a EIA KIT; QuidelOrtho, San Diego, CA), iC3b (cat. # A006; MicroVue iC3b EIA Kit; QuidelOrtho), C5a (cat. # A025; MicroVue C5a EIA Kit; QuidelOrtho), C4d (Compl C4d RUO; Svar Life Science, Malmö, Sweden), terminal complement complexes sC5b-9 (TCC), (cat. # HK 328-01; Hycult Biotech, Uden, the Netherlands). All ELISA analyses were performed according to the respective manufacturer´s instructions. EDTA plasma samples were frozen at -80°C within 2 hours of sampling.

### 2.4 Phenotypic characterization of lymphocytes

Peripheral venous blood samples were collected from HAE-C1INH patients and controls in EDTA tubes (BD Vacutainer cat. # 367862; BD, Franklin Lakes NJ), stored at room temperature, and analyzed with flow cytometry within 24 hours from the time of sampling. The samples were analyzed as routine clinical samples at the Department of Clinical Immunology and Transfusion Medicine, Skåne University Hospital, Lund. In brief, erythrocytes were lysed using 0.84% ammonium chloride (NH4Cl). Lymphocytes and their subpopulations were identified using fluorochrome-conjugated monoclonal antibodies described in **Supplementary Table S2**. The flow cytometers Navios 10 Beckman Coulter^®^ and Navios 8 Beckman Coulter^®^ (Beckman Coulter, Brea, CA) were used for acquisition. Data were analyzed using Kaluza Analysis Software version 2.1 (Beckman Coulter). Definitions of B and T cell subpopulations are shown in **Supplementary Table S3**. Plasmablast concentration values below the detection level of the method (<0.0001 x 10^9)^, found in three patients and in no controls, were set to 0 to enable calculations. One control had terminal effector cells at <1% of CD4^+^ T cells; this value was set to 0 to enable calculations.

### 2.5 Statistical analyses

Statistical analyses were performed with GraphPad Prism 9.4.1 software (GraphPad Software, San Diego, CA). The Mann-Whitney U test was used for two-group comparisons. Correlations were determined by Spearman’s correlation test. Results were considered statistically significant at *p* < 0.05.

### 2.6 Ethical approval

The study was approved by the Swedish Ethical Review Authority (2019–01623 and 2020-05106) and conducted in accordance with the amended Declaration of Helsinki. Patients and controls were included after written informed consent.

## 3 Results

### 3.1 Patient characteristics and demographics

The median age was 50 years (range 21-78) for the patients and the female to male ratio was 69% females (n = 11) vs 31% males (n = 5). The controls were matched for age (+/- 5 years) and sex. At the time of sampling, four (25%) of the HAE-C1INH patients were treated with long-term prophylaxis (LTP); two were treated with attenuated androgens (i.e., danazol), one with plasma-derived C1INH and one with the small molecular kallikrein inhibitor berotralstat. High disease activity was defined as ≥ 2 angioedema attacks per month. Seven of the patients were defined as having high disease activity (HAE^high^) and nine as having low disease activity (HAE^low^) (defined as < 2 angioedema attacks per month). All four patients who were treated with LTP were defined as HAE^low^. All patients and controls had normal leukocyte concentrations (including differential counts). For details, see **Supplementary Table S1**.

### 3.2 Complement proteins and corresponding fragments

To investigate the levels of complement factors and the degree of complement activation in patients with HAE-C1INH, analysis of C1INH, C4, C3 and the complement fragments C4d, C3a, iC3b, C3d, C5a, and TCC was performed. In addition, the C4d/C4 ratio was calculated.

**Figure 1:**
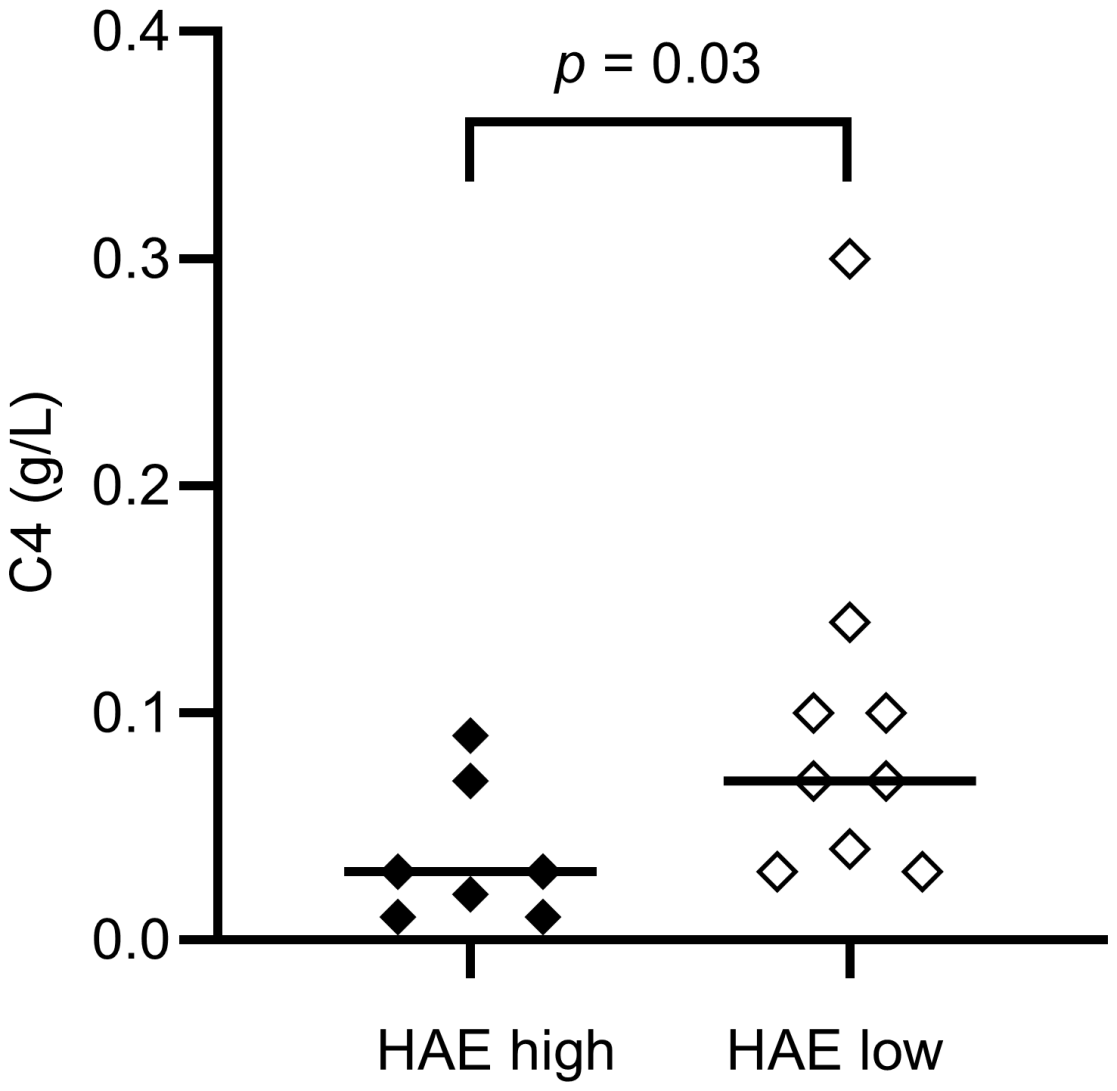
Serum C4 concentrations in HAE-C1INH patients with high disease activity (HAE^high^, defined as ≥ 2 angioedema attacks per month) and low disease activity (HAE^low^, defined as < 2 angioedema attacks per month), respectively. The Mann-Whitney U test was applied for group comparisons.

Levels of complement proteins and fragments in the patient and control cohorts are compared in **Table 1A**, and comparisons of results between HAE^high^ and HAE^low^ are shown in **Table 1B**. As expected, C4 and C1INH levels were lower in HAE-C1INH patients compared to controls (both: *p* < 0.0001). C4 was lower in HAE^high^ compared to HAE^low^ (*p* = 0.03, **Figure 1**). There was no difference in the level of C4d comparing patients and controls, but the C4d/C4 ratio was higher in patients (*p <*0.0001), consistent with increased C4 consumption in HAE-C1INH.

**Table 1A:** Complement and corresponding fragments in HAE-C1INH patients and matched controls.

	HAE-C1INH patients (n=16)	Matched controls (n =16)	*P*-value
C1INH (g/L; ref. 0.20 – 0.38)	0.06 (0.04 – 0.10)	0.27 (0.24 – 0.31)	**<0.0001**
C4 (g/L; ref. 0.13 – 0.39)	0.06 (0.03 – 0.10)	0.24 (0.21 – 0.28)	**<0.0001**
C4d (mg/L)	0.67 (0.36 – 0.75)	0.70 (0.24 – 1.06)	0.51
C4d/C4 ratio	0.009 (0.008 – 0.018)	0.003 (0.001 – 0.005)	**<0.0001**
C3 (g/L; ref. 0.76 - 1.77)	1.07 (0.92 – 1.19)	0.98 (0.93 – 1.09)	0.46
C3a ng/mL	48.8 (36.2 – 78.0)	48.5 (40.2 – 87.8)	0.41
C3d (mg/L; ref. <5)	7.50 (4.78 – 12.13)	3.95 (0.00 – 5.78)	**0.003**
iC3b (µg/mL)	3.67 (3.35 – 4.26)	3.15 (3.08 – 3.26)	**0.007**
C5a (ng/mL)	7.36 (5.58 – 9.69)	6.48 (3.52 – 9.05)	0.48
TCC (mAU/mL)	1593 (1282 – 2092)	1506 (1142 - 1771)	0.47

Data are presented as medians and interquartile ranges. Reference intervals for C1INH, C4, C3 and C3d according to the Department of Clinical Immunology and Transfusion Medicine, Skåne University Hospital, Lund, Sweden. The Mann-Whitney U test was used for comparison of groups. Significant *p*-values in bold.

**Table 1B:** Complement and corresponding fragments in HAE-C1INH patients with high (HAE ^high^) and low (HAE ^low^) disease activity, respectively.

	HAE^high^ (n=7)	HAE^low^ (n=9)	*P*-value
C1INH (g/L; ref. 0.20 – 0.38)	0.04 (0.03 – 0.07)	0.07 (0.06 – 0.14)	0.05
C4 (g/L; ref. 0.13 – 0.39)	0.03 (0.01 – 0.07)	0.07 (0.035 – 0.12)	**0.03**
C4d (mg/L)	0.43 (0.25 – 0.73)	0.73 (0.58 – 0.88)	0.09
C4d/C4 ratio	0.009 (0.008 – 0.043)	0.009 (0.007 – 0.014)	0.84
C3 (g/L; ref. 0.76 – 1.77)	1.07 (0.99 – 1.17)	1.03 (0.905 – 1.24)	0.74
C3a (ng/mL)	39.6 (35.9 – 78.5)	53.2 (37.6 – 81.1)	0.84
C3d (mg/L; ref. <5)	9.5 (7.3 – 14.8)	5.60 (3.60 – 10.25)	**0.03**
iC3b (µg/mL)	4.0 (3.6 – 4.6)	3.4 (3.0 – 4.0)	0.09
C5a (ng/mL)	6.9 (2.7 – 9.3)	7.7 (5.6 – 10.0)	0.56
TCC (mAU/mL)	1688 (1354 – 1878)	1420 (1202 – 2620)	1.0

Data are presented as medians and interquartile ranges. Reference intervals for C1INH, C4, C3 and C3d according to the Department of Clinical Immunology and Transfusion Medicine, Skåne University Hospital, Lund, Sweden. HAE^high^ was defined as ≥2 angioedema attacks per month. HAE^low^ was defined as <2 angioedema attacks per month. The Mann-Whitney U test was used for comparison of groups. Significant *p*-values in bold.

**Figure 2:**
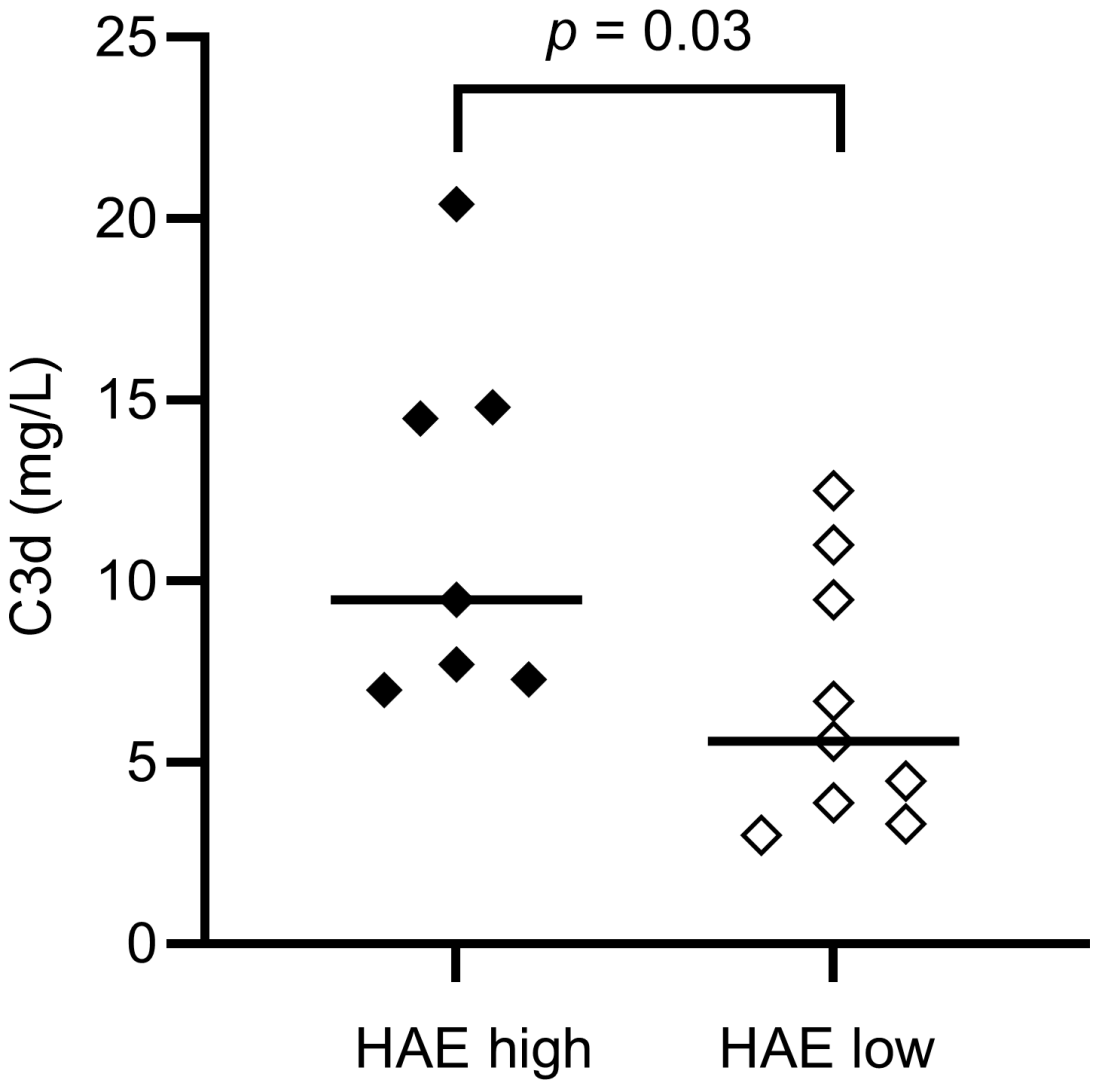
EDTA plasma C3d concentrations in HAE-C1INH patients with high (HAE^high^, defined as ≥2 angioedema attacks per month) and low disease activity (HAE^low^, defined as <2 angioedema attacks per month), respectively. The Mann-Whitney U test was applied for group comparisons.

There was no difference in the levels of C3 or C3a between HAE-C1INH patients and controls, while C3d and iC3b were higher in patients compared to controls (*p* = 0.003 and *p* = 0.007, respectively), indicating a higher degree of complement activation involving the C3 convertase in HAE-C1INH patients. C3d was also higher in HAE^high^ compared to HAE^low^ (*p* = 0.03, **Figure 2**).

### 3.3 Lymphocyte distribution and relation to complement

**Figure 3:**
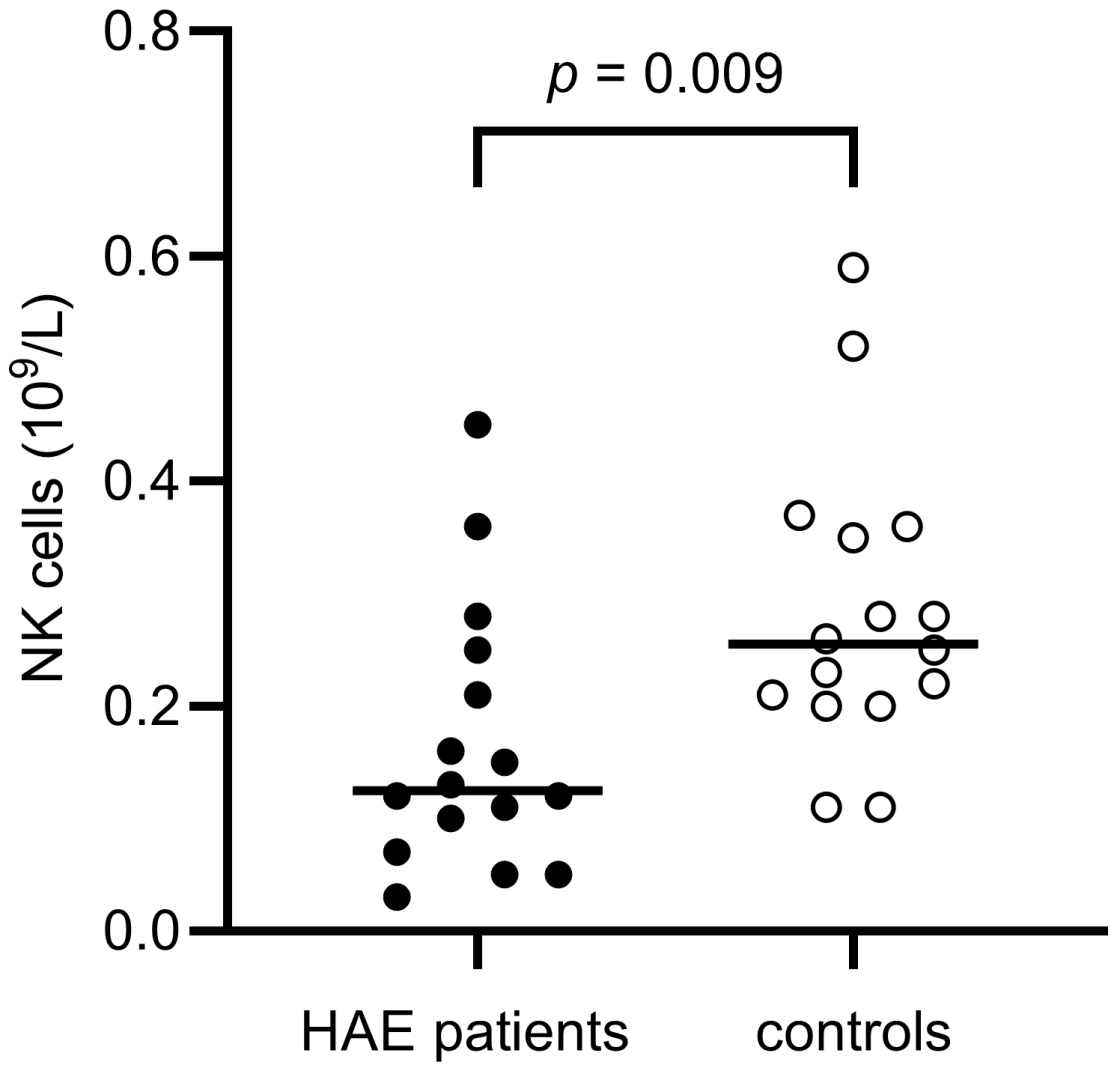
NK cell concentrations in HAE-C1INH patients and matched controls. The Mann-Whitney U test was applied for group comparisons.

**Figure 4:**
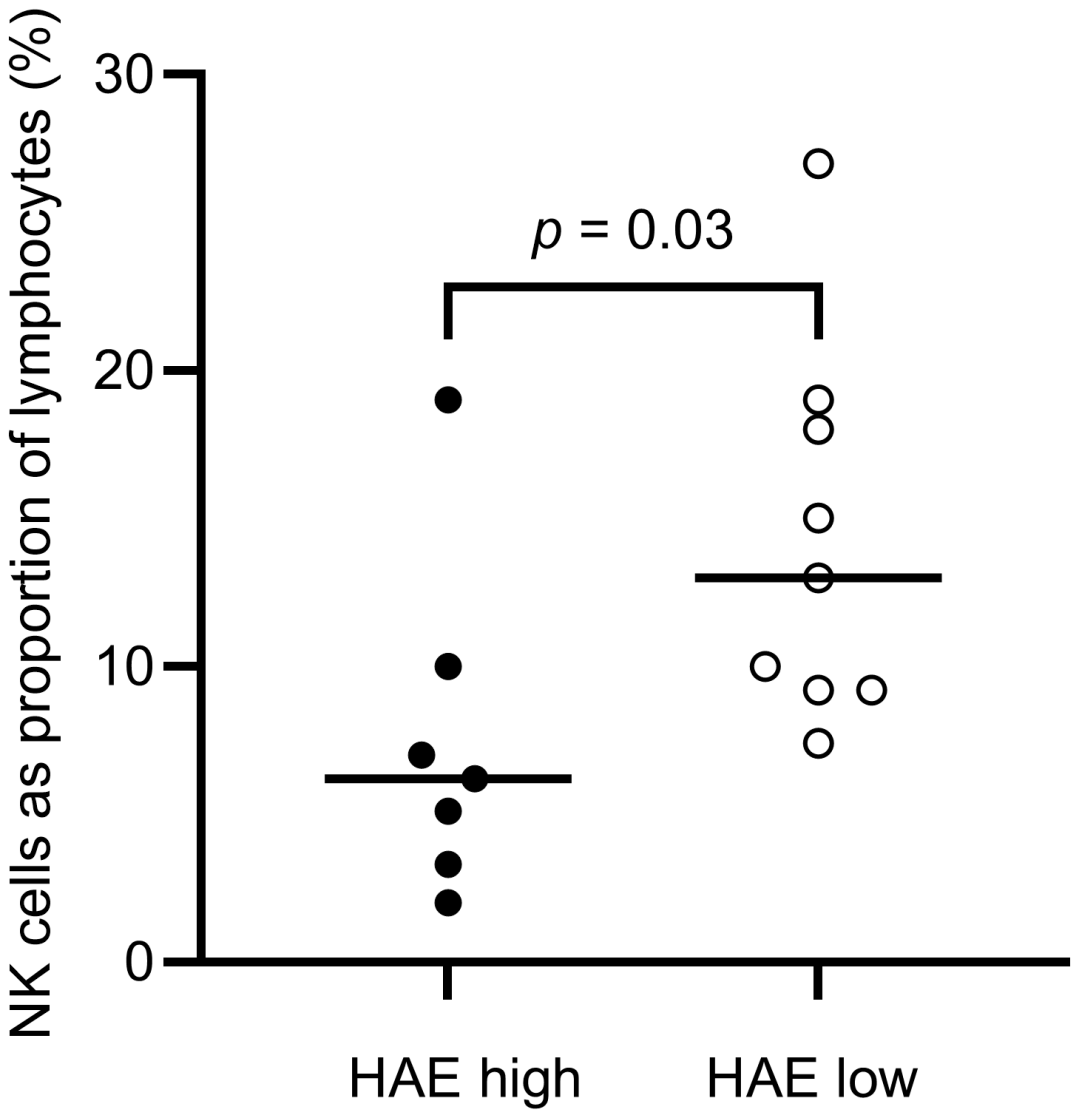
Proportions of NK cells in HAE-C1INH patients with high (HAE^high^, defined as ≥2 angioedema attacks per month) and low disease activity (HAE^low^, defined as <2 angioedema attacks per month), respectively. The Mann-Whitney U test was applied for group comparisons.

The distribution of lymphocytes in the patient and control cohorts are shown in **Table 2**. Total lymphocyte counts did not differ significantly between patients and controls. However, NK cell counts were lower in the HAE-C1INH population (median: 0.13 x 10^9^ cells/L, interquartile range (IQR): 0.08 – 0.24) compared to the control group (median: 0.26 x 10^9^ cells/L, IQR: 0.20-0.36; *p* = 0.009, **Figure 3**). HAE^high^ individuals had a lower proportion of NK cells compared to HAE^low^ (*p* = 0.03, **Figure 4**). There were no other differences regarding lymphocyte concentrations and proportions between HAE^high^ and HAE^low^. Calculations of correlations of lymphocytes and complement were performed for parameters which differed between patients and controls.

**Table 2:** Distribution of lymphocyte populations in HAE-C1INH patients and matched controls.

Phenotype	HAE-C1INH patients (n= 16)	Matched controls (n=16)	*P*-value
Lymphocytes, x 10^9^ cells/L (Ref. 0.82 - 2.60)	1.5 (1.4 - 1.7)	1.8 (1.4 - 2.7)	0.16
NK cells, % of lymphocytes (Ref. 4.2 - 26)	9.6 (6.4 - 17.3)	14.0 (9.6 - 18.0)	0.10
NK cells, x 10^9^ cells/L (Ref. 0.08 – 0.57)	0.13 (0.08 - 0.24)	0.26 (0.20 - 0.36)	**0.009**
B cells, % of lymphocytes (Ref. 5.5 - 20)	11.5 (7.9 - 13.8)	9.9 (7.3 - 13.0)	0.58
B cells, x 10^9^ cells/L (Ref. 0.07 - 0.46)	0.15 (0.12 - 0.19)	0.18 (0.13 - 0.28)	0.19
Naïve, % of B cells (Ref. 32 - 81)	77 (57 - 81)	62 (54 - 70)	0.06
Transitional naïve, % of B cells (Ref. 2.1 - 14)	5.4 (3.8 - 7.5)	4.5 (2.5 - 6.8)	0.19
Pre-switched (marginal zone), % of B cells (Ref. 4.9 - 23)	6.9 (3.8 - 11.8)	10.5 (8.3 - 13.8)	0.07
Class-switched memory cells, % of B cells (Ref. 6.6 - 42)	13.5 (7.5 - 19.8)	17.5 (13.0 - 27.5)	**0.04**
Plasmablasts, % of B cells (Ref. 0.11 - 2.0)	0.29 (0.17 - 0.70)	0.65 (0.50 - 2.3)	**0.01**
Plasmablasts, x 10^9^ cells/L (Ref. 0.0002 - 0.0026)	0.00050 (0.00030 - 0.00088)	0.0016 (0.00060 - 0.0053)	**0.01**
CD21 Low, % of B cells (Ref. 0.64 - 5.1)	2.4 (1.6 - 3.8)	2.7 (1.4 - 3.9)	0.89
T cells, % of lymphocytes (Ref. 61 - 85)	79 (74 - 83)	75 (69 - 78)	0.13
T cells, x 10^9^ cells/L (Ref. 0.60-2.10)	1.15 (1.03 - 1.41)	1.27 (0.98 - 1.97)	0.29
CD4^+^, % of T cells (Ref. 27 - 56)	48 (41 - 54)	51 (43 - 52)	0.66
CD4^+^, x 10^9^ cells/L (Ref. 0.40 - 1.20)	0.77 (0.58 - 0.83)	0.87 (0.61 - 1.40)	0.11
CD8^+^, % of T cells (Ref. 17 - 41)	28 (22 - 31)	26 (20 - 28)	0.16
CD8^+^, x10^9^ cells/L (Ref. 0.18 - 0.90)	0.42 (0.22 - 0.53)	0.42 (0.35 - 0.54)	0.55
Naïve, % of CD4^+^ (Ref. 7.4 - 57)	49 (39 - 54)	52 (43 - 60)	0.16
Terminal effector, % of CD4^+^ (Ref. 0.5 - 15)	6.80 (2.85 - 14.75)	6.05 (1.78 - 12.00)	0.54
Central memory, % of CD4^+^ (Ref. 18 - 68)	28 (17 - 44)	28 (20 - 38)	0.89
Th1, % of central memory cells (Ref. 19 - 41)	25 (20 - 32)	31 (25 - 37)	**0.03**
Th2, % of central memory cells (Ref. 12 - 44)	24 (21 - 39)	23 (18 - 26)	0.35
Th17, % of central memory cells (Ref. 19 - 34)	26 (21 - 33)	22 (20 - 25)	0.13
Effector memory, % of CD4^+^ (Ref. 10 - 36)	16 (14 - 17)	13 (6 - 17)	0.09
Th1, % of effector memory cells (Ref. 12 - 44)	31 (25 - 34)	34 (30 - 50)	0.07
Th2, % of effector memory cells (Ref. 11 - 51)	27 (18 - 35)	20 (12 - 24)	**0.03**
Th17, % of effector memory cells (Ref. 13 - 27)	21 (15 - 27)	18 (14 - 21)	0.11
Naïve, % of CD8^+^ cells (Ref. 5.9 - 68)	45 (33 - 53)	47 (40 - 62)	0.27
Terminal effector, % of CD8^+^ (Ref. 9.2 - 61)	33 (25 - 46)	29 (15 - 43)	0.22
Central memory, % of CD8^+^ (Ref. 0.4 - 16)	10 (6 - 21)	12 (6 - 17)	0.77
Effector memory, % of CD8^+^ (Ref. 19 - 65)	7 (4 - 13)	7 (2 - 10)	0.38
T-regs, % of CD4^+^ (Ref. 1.7 - 6.1)	2.7 (2.0 - 3.0)	2.7 (2.1 - 3.3)	0.96
HLA-DR, % of T-regs (Ref. 31 - 73)	48 (38 - 57)	46 (39 - 52)	0.59
CD4/CD8 ratio (Ref. 0.90 - 3.20)	1.7 (1.4 - 2.1)	1.8 (1.7 - 2.6)	0.20

Data are presented as medians and interquartile ranges. Reference intervals for lymphocyte populations according to the Department of Clinical Immunology and Transfusion Medicine, Skåne University Hospital. The Mann-Whitney U test was used for comparisons of groups. Significant *p*-values in bold.

**Figure 5:**
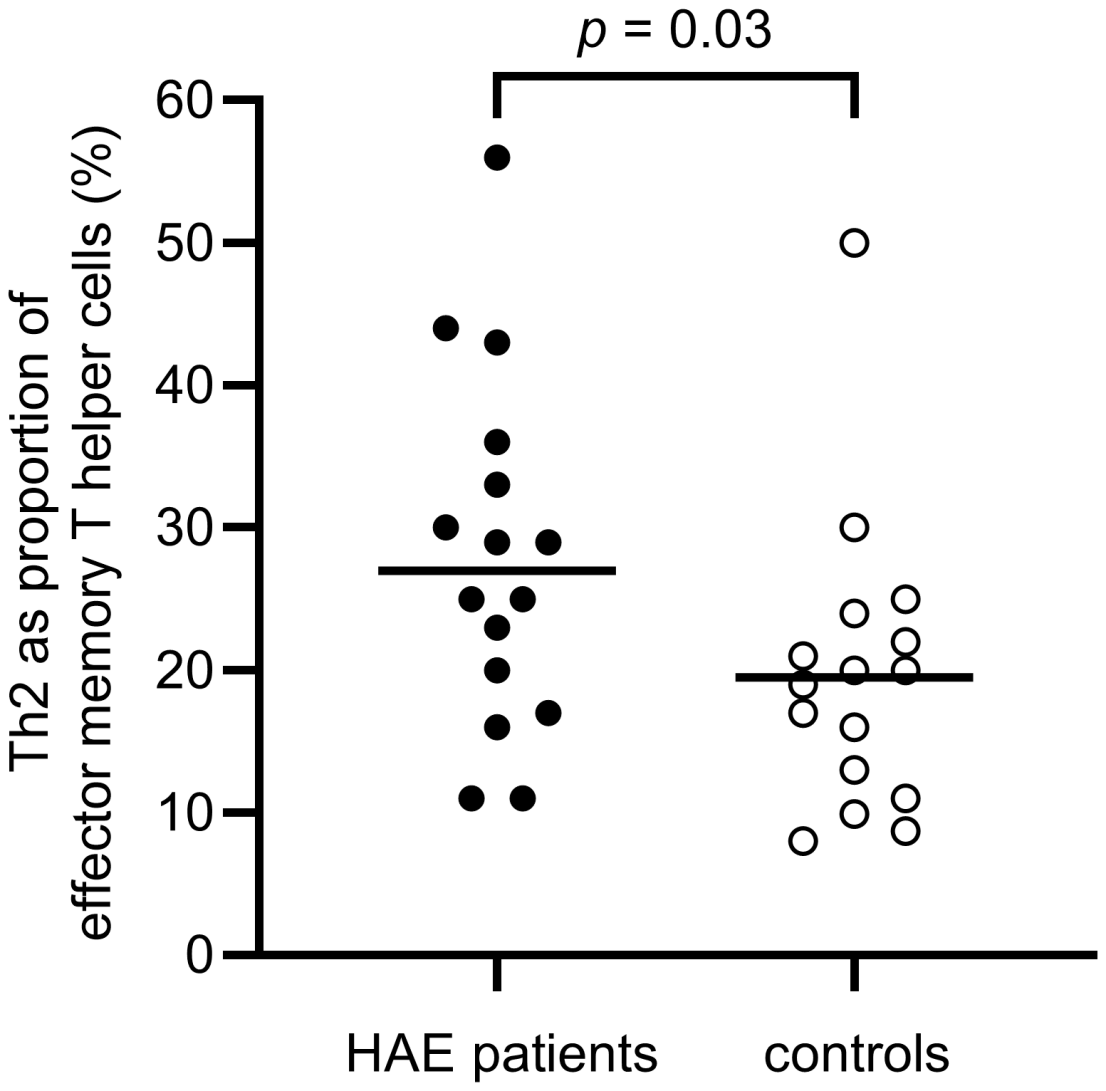
Proportions of Th2 of effector memory T helper cells in HAE-C1INH patients and matched controls. The Mann-Whitney U test was applied for group comparisons.

**Figure 6:**
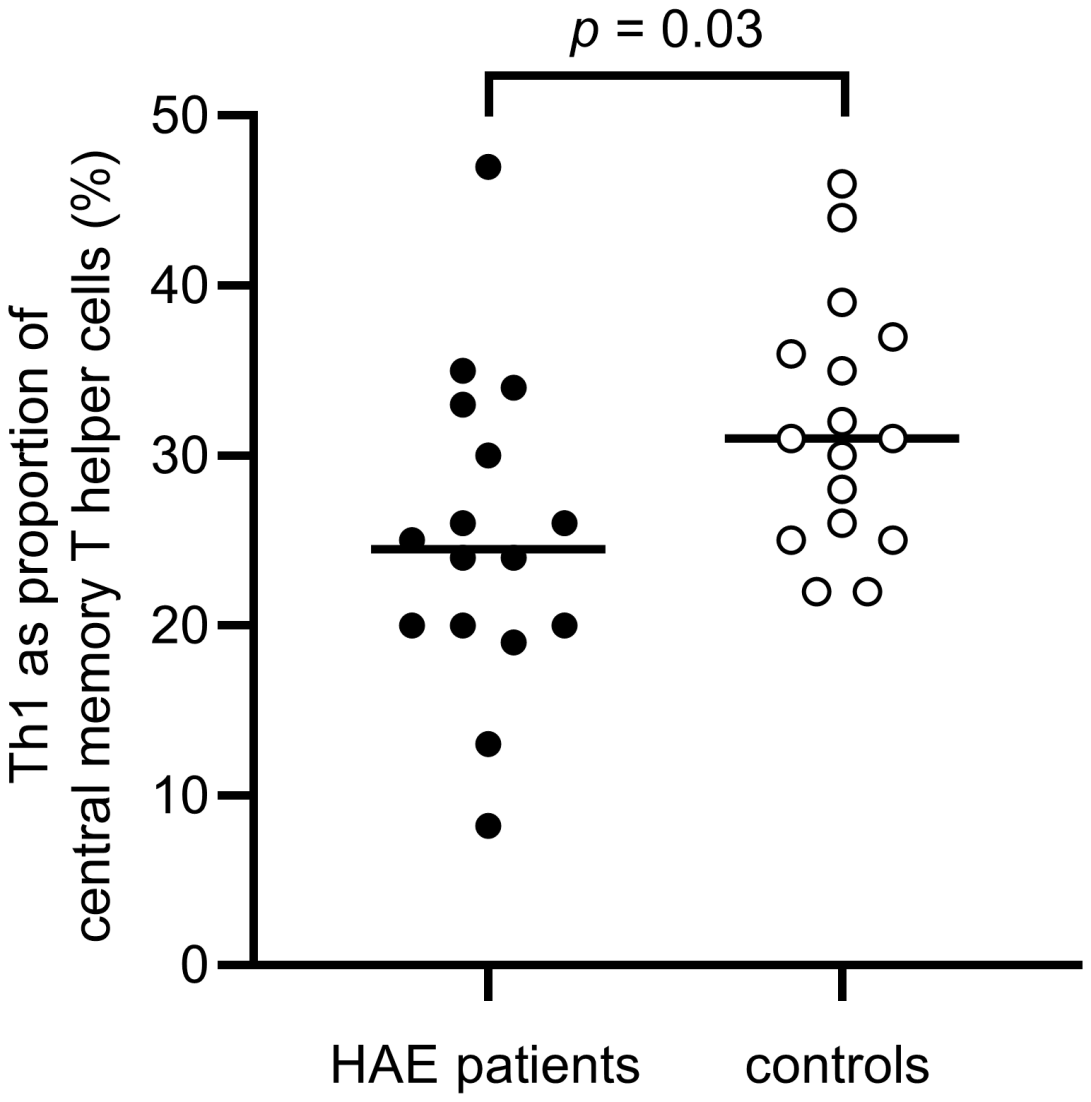
Proportions of Th1 of central memory T helper cells in HAE-C1INH patients and matched controls. The Mann-Whitney U test was applied for group comparisons.

**Figure 7:**
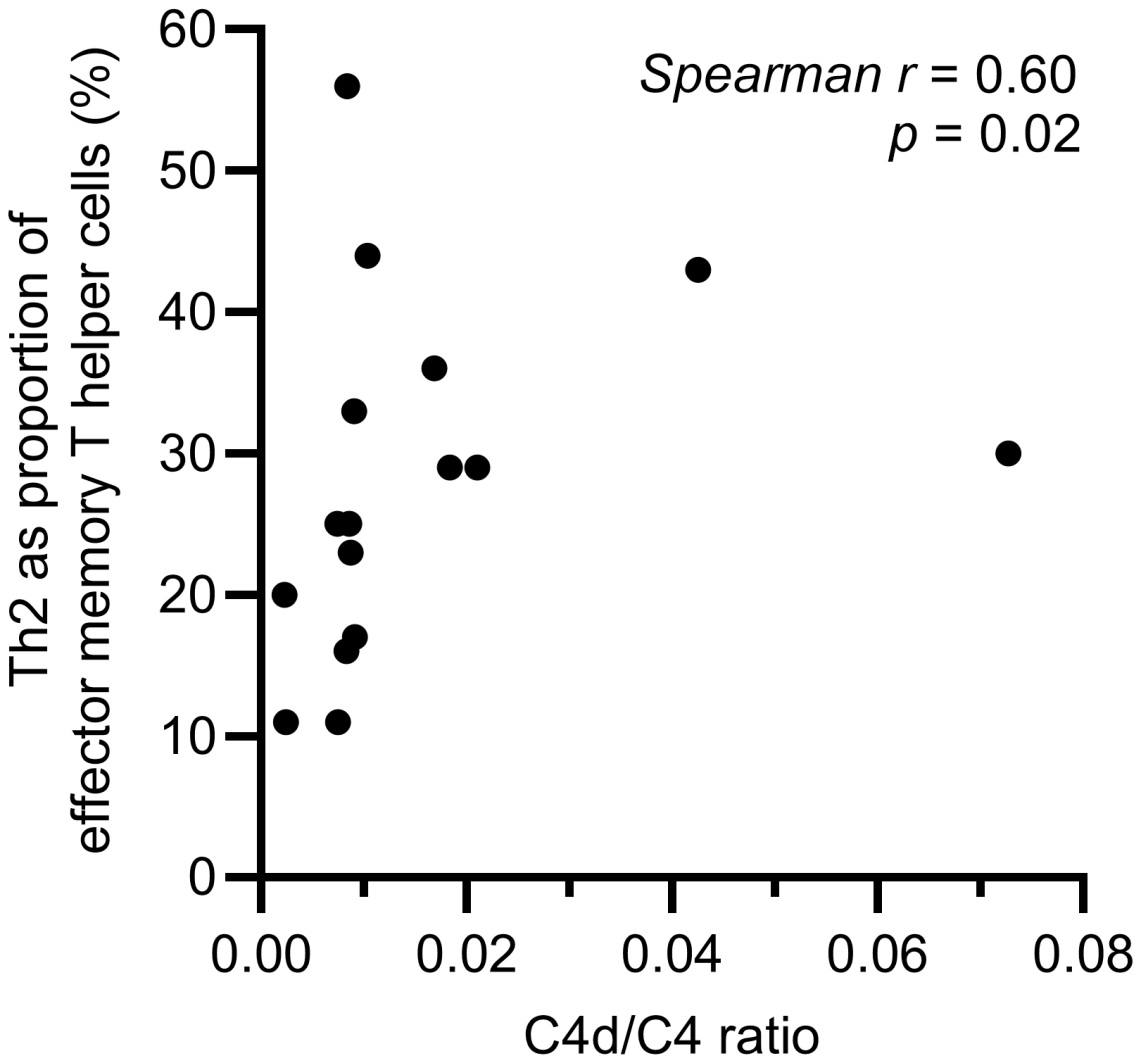
Correlation between C4d/C4 ratio and Th2 effector memory T helper cells in HAE-C1INH patients. The correlation was determined using the Spearman rank correlation test.

A difference in the balance between Th1 and Th2 cells was observed, with a significantly higher proportion of Th2 cells in the effector memory T helper cell population among HAE-C1INH patients compared to controls (*p* = 0.03, **Figure 5**). The proportion of Th1 cells in the central memory T helper cell population was lower in patients with HAE-C1INH compared to controls (*p* = 0.03, **Figure 6**). In HAE-C1INH patients, Th2 effector memory T helper cells also correlated with C4d/C4 ratios (*p* = 0.02, **Figure 7**). No other correlations between lymphocyte populations and complement were found.

**Figure 8:**
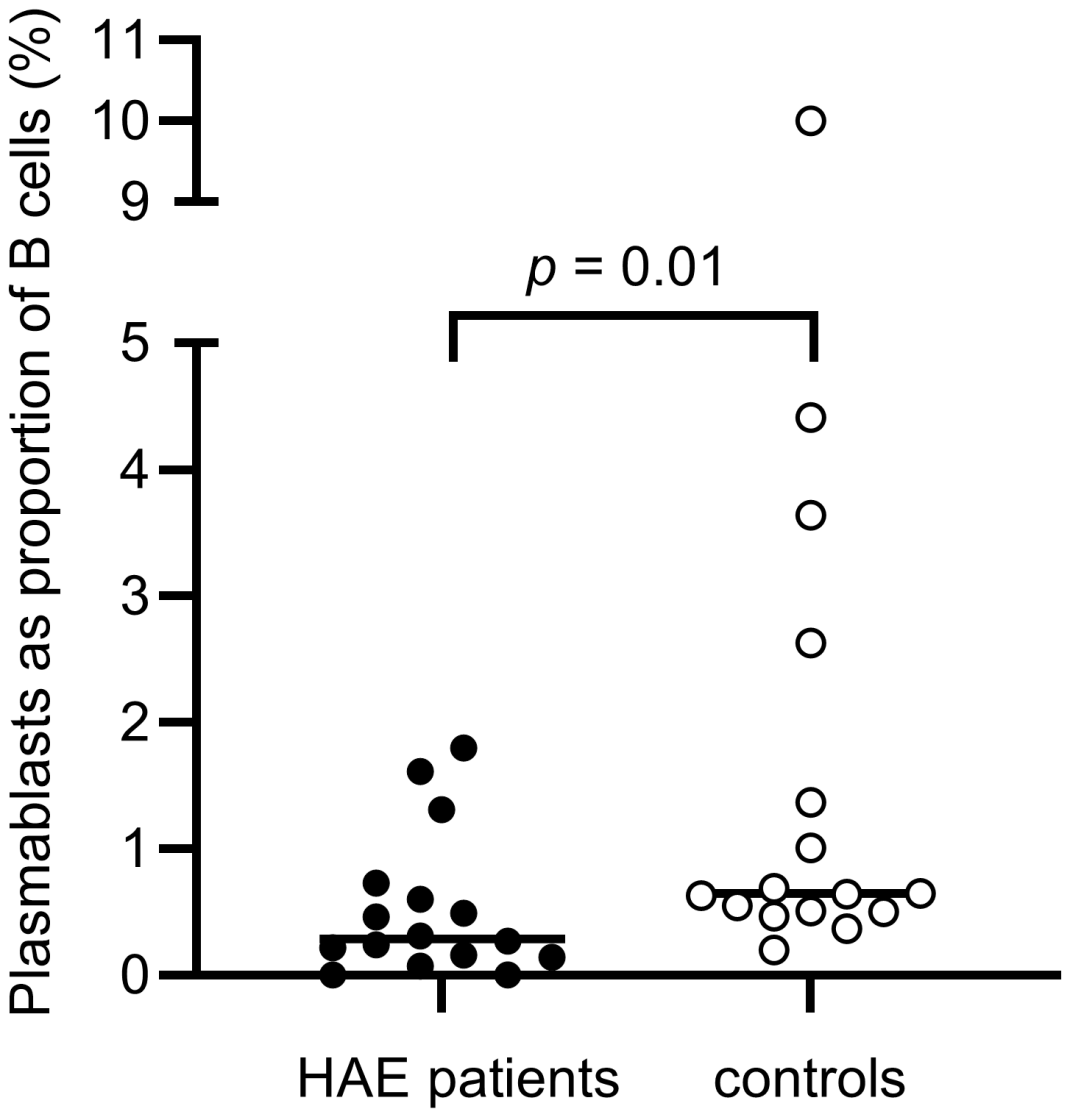
Proportions of plasmablasts of B cells in HAE-C1INH patients and matched controls. The Mann-Whitney U test was applied for group comparisons.

**Figure 9:**
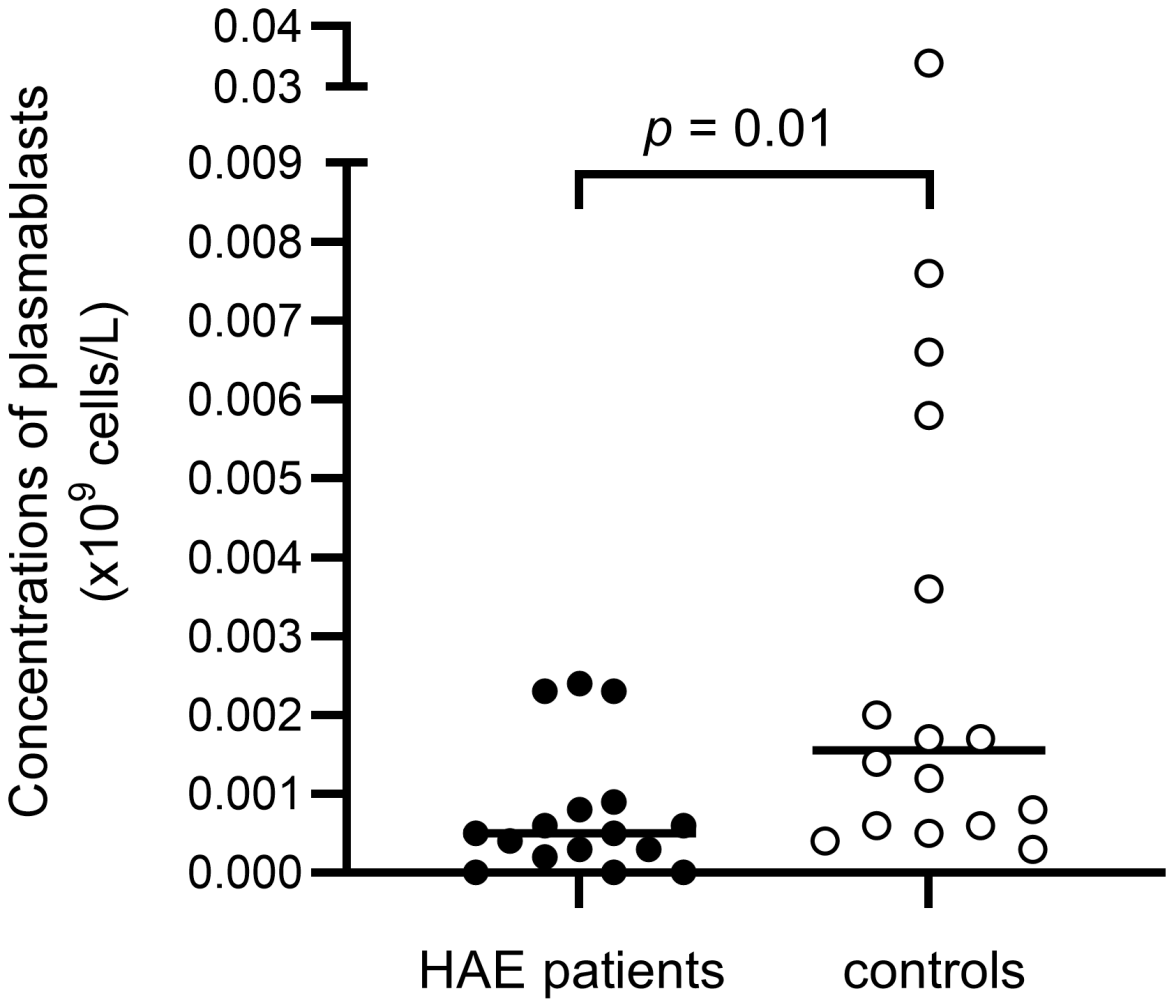
Concentrations of plasmablasts in HAE-C1INH patients and matched controls. The Mann-Whitney U test was applied for group comparisons.

There were no differences in total B cell counts or percentages between patients and controls, but subpopulations were differently distributed, with HAE-C1INH patients having less mature B cells: HAE-C1INH patients had a lower proportion of class-switched memory B cells (*p* = 0.04), and plasmablasts were lower in both frequency (*p* = 0.01) and total count (*p* = 0.01) in HAE patients compared to controls (**Figures 8**, **9**).

The three patients with autoimmune disease (of which two were HAE^high^ and one was HAE^low^) had higher proportions of effector memory T helper cells and lower proportions of naïve CD8^+^ T cells both compared to patients without autoimmune disease (*p* = 0.01 and *p* = 0.02, respectively) and compared to all healthy controls (*p* = 0.02 and *p* = 0.008, respectively). Meanwhile, the same lymphocyte groups in the rest of the patients (without autoimmune disease) were not different from the proportions in all controls. Also, the three patients with autoimmune disease had lower proportions of pre-switched B cells (*p* = 0.02) compared to patients without autoimmune disease, as well as compared to all controls (*p* = 0.004). Compared to all controls, the proportion of naïve B cells was higher (*p* = 0.008), the proportion of class-switched memory B cells was lower (*p* = 0.03), and plasmablasts were lower both in proportion (*p* = 0.01) and total count (*p* = 0.02) in patients with autoimmune disease. In comparison to matched controls (n=3), no corresponding differences were statistically significant.

The differences between HAE-C1INH patients and matched controls were similar when the three patients with autoimmune disease and their matched controls were omitted from the calculations, as compared to the differences observed for the whole patient and control groups. Lower NK cells and a skewed T helper cell balance were still present, as well as lower proportion of plasmablasts. However, the difference was no longer significant regarding proportions of class-switched memory B cells. Even when omitting the three patients with autoimmune disease, HAE^high^ had a lower proportion of NK cells compared to HAE^low^ (*p* = 0.01).

T helper cell balance (Th1 vs. Th2) and NK cell counts were not different in patients with autoimmune disease compared to the rest of the patients.

## 4 Discussion

Although an increased prevalence of autoimmune disease, predominantly SLE, has been recognized in HAE-C1INH, few studies have investigated lymphocytes and other aspects of the immune system in this disease. Therefore, we analyzed complement activation biomarkers and lymphocyte subpopulations in HAE-C1INH patients and healthy, matched controls. These parameters were also compared in HAE-C1INH patients with high (HAE^high^) or low disease activity (HAE^low^), and the three patients with autoimmune disease were compared to the rest of the patients and to controls.

Patients with HAE-C1INH had a lower blood concentration of NK cells compared to the control group. In addition, HAE^high^ patients had a lower proportion of NK cells compared to HAE^low^ patients. A decrease in peripheral NK cell counts has been described in SLE, as well as in other autoimmune diseases like rheumatoid arthritis and diabetes type 1 (10–14). NK cells play a significant role in inflammation and immune regulation and bridge between innate and adaptive immunity (15). There is also increasing evidence for regulation of T cells by NK cells (16, 17).

Interestingly, our study demonstrates a difference in the balance between Th1 and Th2 cells with a predominance of Th2 cells in individuals with HAE-C1INH compared to healthy controls, as we demonstrated a higher proportion of Th2 among effector memory T helper cells and a lower proportion of Th1 among central memory T helper cells. The Th2 emphasis might reflect that HAE-C1INH patients are prone to type 2 inflammatory diseases, for example allergy, asthma, and autoimmune diseases. In line with this, our previous register-based study showed a two-fold higher prevalence of allergy, asthma, and atopic dermatitis, as well as a higher risk of autoimmune diseases in HAE-C1INH patients (3). The findings of a decrease in NK cell counts in combination with a Th2-skewed T helper cell balance in HAE-C1INH patients are interesting. NK cells are characterized as group 1 innate lymphoid cells (ILC), producing IFN-γ, and promoting a type 1 inflammatory response. Hence, NK cells might be considered a counterbalance to group 2 ILCs. IFN-γ has been shown to suppress type 2 inflammation, suggesting that NK cell deficiency might be a relevant feature of type 2 inflammatory diseases like atopic dermatitis (18, 19). Previous studies have demonstrated that NK cells and IFN-γ can restrain ILC2 responses both *in vitro* and during allergic inflammation (20). Regarding cytokine profile, a recent study of HAE-C1INH patients in remission found increased serum levels of the Th2 signature cytokine IL-4 and other proinflammatory cytokines, while IFN-γ concentrations were similar in patients with HAE-C1INH and healthy controls (21). Regarding B cell subsets, we found a less mature profile, with a tendency towards a higher proportion of naïve B cells (*p* = 0.06), and a significantly lower proportion of class-switched memory B cells (*p* = 0.04) in HAE-C1INH patients compared to controls. Transitional naïve B cells are key players in autoimmune disease and are elevated in SLE patients (22–24). There are also studies indicating that transitional naïve B cells cause early loss of B cell tolerance in SLE patients (25). Surprisingly, plasmablasts were lower both in frequency and total counts in patients compared to controls. This contrasts to the situation in SLE, where increased plasmablasts have been reported (26).

HAE-C1INH patients have low levels of C4 due to chronic activation of the classical complement pathway, also leading to low complement function. The classical pathway is important for the removal of immune complexes and dying cells, and low function of the classical pathway is associated with development of autoimmune diseases, mainly SLE (7, 27). Considering the connection between the classical complement activation pathway and the risk of autoimmunity, it is interesting that we found a correlation between on the one hand C4d/C4 ratio, which reflects the degree of complement consumption, and on the other hand the proportion of Th2 cells among effector memory Th cells, as Th2 cells are known to play important roles both in type 2 inflammatory conditions and in autoimmune pathogenesis. Prospective studies of HAE-C1INH patients are warranted, to investigate the potential prognostic information of e.g., C4d/C4 ratios and Th2 cells on the risk of developing type 2 inflammatory diseases and autoimmunity.

We found an increase in the C3 cleavage products C3d and iC3b in HAE-C1INH patients, indicating increased C3 activation. These findings are in line with results from previous studies on HAE-C1INH patients (21, 28). However, there was no difference in C3a levels between patients and controls, which may reflect its rapid elimination from the circulation. The finding of higher C3d in HAE^high^ compared to HAE^low^ should be further investigated regarding C3d as a potential biomarker of disease activity.

The terminal pathway activation markers C5a and TCC were not significantly higher in HAE-C1INH patients compared to controls, which is in line with previous studies on HAE-C1INH patients that showed increased TCC only during HAE attacks (21, 28, 29). Thus, during baseline conditions, HAE-C1INH patients are not likely to be exposed to the proinflammatory effects of terminal pathway complement activation.

Regarding the relationship between complement consumption and the dysregulated adaptive immunity in HAE-C1INH, little is known about the influence of complement on NK cells and other ILCs while there is substantial evidence for an impact on the regulation of both T and B cells (30–32). In HAE-C1INH, it is possible that not only the chronic activation of the classical pathway with associated low complement function, but also the production of C3 fragments may act in concert to affect immune regulation (32, 33).

The three patients with manifest autoimmune disease showed a more pronounced B cell immaturity and a tendency to an increase in T cell memory proportions compared to the rest of the HAE-C1INH patients, but did not differ from the rest of the patients regarding NK cells or T helper cell balance. Possibly, the detected differences are a result of the autoimmune diseases *per se.* This could be further investigated in a larger cohort of HAE-C1INH patients with and without manifest autoimmune disease.

Limitations of our study are the low numbers of patients, and that few male patients were included.

## 5 Conclusions

We demonstrate altered lymphocyte distributions in HAE-C1INH, with lower NK cells, a Th2 predominance, and less mature B cells compared to matched healthy controls. The findings are interesting, considering the increased risk of both autoimmune disease and allergy associated with HAE-C1INH. Further studies are needed to investigate mechanisms connecting complement consumption to effects on lymphocyte distribution and function. Such studies could have an impact on the choice of prophylaxis offered to HAE-C1INH patients, of which there is now a wide spectrum with different targets. Prophylactic medication with distinct targets has the potential of providing an improved balance of the immune system, thereby decreasing the risk of comorbid conditions associated with HAE-C1INH.

## Data Availability

The raw data supporting the conclusions of this article will be made available by the authors, without undue reservation.
