# Supplementary material for: Th2 predominance and decreased NK cells in patients with hereditary angioedema
Source: Front Immunol. 2025 May 14;16:1536128. doi: 10.3389/fimmu.2025.1536128 (PMC12116338; doi:10.3389/fimmu.2025.1536128)
Supplement: Supplementary file 1 [file DataSheet1.docx]

Supplementary Material

# Supplementary Tables

**Supplementary Table S1. Characteristics and demographics of HAE-C1INH patients with high (HAE^high^) and low (HAE^low^) disease activity, respectively.**

|  | **HAE^high^ (n=7)** | **HAE^low^ (n=9)** |
| --- | --- | --- |
| Female, n (%) | 5 (71) | 6 (67) |
| Age, years; median (IQR) | 44 (24 – 54) | 55 (30 – 63) |
| No long-term prophylaxis (LTP), n (%) | 7 (100) | 5 (56) |
| LTP: androgens, n (%) | 0 | 2 (22) |
| LTP: C1inh substitution, n (%) | 0 | 1 (11) |
| LTP: Berotralstat, n (%) | 0 | 1 (11) |
| B-Platelets x 10^9^/L (ref. 165 – 387) median (IQR) | 258 (210 – 273) | 267 (218 – 321) |
| B-WBC x 10^9^/L (ref. 3.5 – 8.8)  median (IQR) | 7.0 (5.8 – 7.9) | 6.6 (6.1 – 8.2) |
| B-Neutrophils x 10^9^/L (ref. 1.7 – 8.0) median (IQR) | 4.5 (3.3 – 5.2) | 4.4 (3.7 – 5.7) |
| B-Eosinophils x 10^9^/L, (ref. <0.7) median (IQR) | 0.1 (0 – 0.4) | 0 (0 – 0.2) |
| B-Basophils x 10^9^/L, (ref. < 0.3) median (IQR) | 0.0 | 0.0 |
| B-Lymphocytes x 10^9^/L, (ref. 1.1– 4.8) median (IQR) | 1.8 (1.6 – 1.9) | 1.4 (1.4 – 2.1) |
| B-Monocytes x 10^9^/L, (ref. <1.1) median (IQR) | 0.5 (0.4 – 0.5) | 0.5 (0.5 – 0.7) |

HAE^high^ is defined as $\geq$ 2 angioedema attacks per month and HAE^low^ is defined as < 2 angioedema attacks per month. IQR; interquartile range, LTP; long-term prophylaxis, WBC; white blood cell count. Reference intervals for leukocytes according to the Department of Clinical Chemistry, Region Skåne.

**Supplementary Table S2. Antibody panel**

| **Antibody mix** | | **Fluorochrome** | **Supplier/art. No** | | **Clone** |
| --- | --- | --- | --- | --- | --- |
| T, B and NK cells | |  |  | |  |
| CD19 | | PC5.5 | Beckman Coulter® B49211 | | J3-119 |
| CD16 | | PE | Beckman Coulter® A07766 | | 3G8 |
| CD56 | | PE | Beckman Coulter® A07788 | | N901 |
| CD3 | | APC -Alexa Fluor 750 | Beckman Coulter® A94680 | | UCHT1 |
| CD8 | | APC-Alexa Fluor 700 | Beckman Coulter® B49181 | | B9.11 |
| CD45 | | ECD | Beckman Coulter® A07784 | | J33 |
| CD4 | | APC | Beckman Coulter® IM2468 | | 13B.8.2 |
| **Naïve, transitional and memory B cells and plasmablasts** | | | | | |
| DuraClone IM B cells tube:  IgD, CD21, CD19, CD27, CD24, CD38, IgM, CD45 | | | Beckman Coulter® B53318 | |  |
| **Naïve and memory T cells** | | |  | |  |
| CD45 ECD | | | Beckman Coulter® A07784 | | J33 |
| CD4 APC | | | Beckman Coulter® IM2468 | | 13B.8.2 |
| CD8 APC-Alexa Fluor 700 | | | Beckman Coulter® B49181 | | B9.11 |
| CD3 PC -Alexa Fluor 750 | | | Beckman Coulter® A94680 | | UCHT1 |
| CD45RA FITC | | | BioLegend 304106 | | HI100 |
| CD45RO PE | | | Beckman Coulter® A07787 | | HIT2 |
| CD197 PerCP/Cy5.5 | | | BD 561144 | | 150503 |
| **Naïve, memory and Th1, Th2, Th17** | | | | | |
| CD45 | ECD Beckman Coulter^®^ A07784 | | | J33 | |
| CD4 | APC Beckman Coulter^®^ IM2468 | | | 13B.8.2 | |
| CD8 | APC-Alexa Fluor 700 Beckman Coulter^®^ B49181 | | | B9.11 | |
| CD3 | PC -Alexa Fluor 750 Beckman Coulter^®^ A94680 | | | UCHT1 | |
| CD45RA | FITC BioLegend 304106 | | | HI100 | |
| CD183 | PE BioLegend 353706 | | | G025H7 | |
| CD197 | PerCP/Cy5.5 BD 561144 | | | 150503 | |
| CD196 | PC7 BioLegend 353418 | | | G034E3 | |
| **T regs** |  | | |  | |
| CD45 | ECD Beckman Coulter^®^ A07784 | | | J33 | |
| CD127 | Alexa Fluor 647 BD 558598 | | | HIL-7R-M21 | |
| CD45RO | Alexa Fluor 700 BioLegend 304218 | | | UCHL-1 | |
| CD3 | APC -Alexa Fluor 750Beckman Coulter^®^ A94680 | | | UCHT1 | |
| CD4 | APC Beckman Coulter^®^ IM2468 | | | 13B.8.2 | |
| CD25 | PE BD 341011 | | | M-A251 | |
| CD194 | PC5.5 BD 560726 | | | 1G1 | |
| HLA-DR | PC7 Beckman Coulter^®^ B49180 | | | Immu357 | |

**Supplementary Table S3. Definitions of lymphocytes and subpopulations**

| Phenotype | Cell surface markers |
| --- | --- |
| **NK cell** | CD3^-^ CD56^+^ CD16^+^ |
| **B cell (CD19^+^)** |  |
| Naïve B cells of B cells | IgD^+^ CD27^-^ |
| Pre-switch of B cells (marginal zone B cells) | IgD^+^ CD27^+^ |
| Class-switched memory cells of B cells | IgD^-^ CD27^+^ |
| Transitional B cells of B cells | CD38^+^ CD24^+^ IgD^+^ CD27^-^ |
| CD21 low of B cells | CD38^-^ CD21^low^ |
| **T cell (CD3^+^)** |  |
| Naïve CD4^+^ cells | CD45RA^+^ CD197^+^ |
| Terminal effector cells of CD4^+^ | CD45RA^+^ CD197^-^ |
| Central memory cells of CD4^+^ | CD45RA^-^ CD197^+^ |
| Effector memory cells of CD4^+^ | CD45RA^-^ CD197^-^ |
| Naive CD8^+^ cells | CD45RA^+^ CD197^+^ |
| Terminal effector cells of CD8^+^ | CD45RA^+^ CD197^-^ |
| Central memory cells of CD8^+^ | CD45RA^-^ CD197^+^ |
| Effector memory cells of CD8^+^ | CD45RA^-^ CD197^-^ |
| T-regs of CD4^+^ T cells | CD3^+^ CD4^+^ CD25^+^ CD127^-^ CD45RO^+^ CD194^+^ |
| Activated T-regs of T-regs | CD3^+^ CD4^+^ CD25^+^ CD127^-^ CD45RO^+^ CD194^+^ HLA-DR^+^ |
| Th1 of central memory cells | CD3^+^ CD4^+^ CD45RA^-^ CD197^+^ CD183^+^ CD196^-^ |
| Th2 of central memory cells | CD3^+^ CD4^+^ CD45RA^-^ CD197^+^ CD183^-^ CD196^-^ |
| Th17 of central memory cells | CD3^+^ CD4^+^ CD45RA^-^ CD197^+^ CD183^-^ CD196^+^ |
| Th1 of effector memory cells | CD3^+^ CD4^+^ CD45RA^-^ CD197^-^ CD183^+^ CD196^-^ |
| Th2 of effector memory cells | CD3^+^ CD4^+^ CD45RA^-^ CD197^-^ CD183^-^ CD196^-^ |
| Th17 of effector memory cells | CD3^+^ CD4^+^ CD45RA^-^ CD197^-^ CD183^-^ CD196^+^ |
